# A community-based resource for automatic exome variant-calling and annotation in Mendelian disorders

**DOI:** 10.1186/1471-2164-15-S3-S5

**Published:** 2014-05-06

**Authors:** Margherita Mutarelli, Veer Singh Marwah, Rossella Rispoli, Diego Carrella, Gopuraja Dharmalingam, Gennaro Oliva, Diego di Bernardo

**Affiliations:** 1 Telethon Institute of Genetics and Medicine, Via P. Castellino 111, 80131 Naples, Italy; 2 Fondazione Biology For Medicine, Via P. Castellino 111, 80131 Naples, Italy; 3 Institute for high performance computing and networking -CNR, Via P. Castellino 111, 80131 Naples, Italy; 4 Department of Electrical Engineering and Information Technology, Università degli Studi di Napoli Federico II, Via Claudio 21, 80125 Naples, Italy

## Abstract

**Background:**

Mendelian disorders are mostly caused by single mutations in the DNA sequence of a gene, leading to a phenotype with pathologic consequences. Whole Exome Sequencing of patients can be a cost-effective alternative to standard genetic screenings to find causative mutations of genetic diseases, especially when the number of cases is limited. Analyzing exome sequencing data requires specific expertise, high computational resources and a reference variant database to identify pathogenic variants.

**Results:**

We developed a database of variations collected from patients with Mendelian disorders, which is automatically populated thanks to an associated exome-sequencing pipeline. The pipeline is able to automatically identify, annotate and store insertions, deletions and mutations in the database. The resource is freely available online http://exome.tigem.it. The exome sequencing pipeline automates the analysis workflow (quality control and read trimming, mapping on reference genome, post-alignment processing, variation calling and annotation) using state-of-the-art software tools. The exome-sequencing pipeline has been designed to run on a computing cluster in order to analyse several samples simultaneously. The detected variants are annotated by the pipeline not only with the standard variant annotations (e.g. allele frequency in the general population, the predicted effect on gene product activity, etc.) but, more importantly, with allele frequencies across samples progressively collected in the database itself, stratified by Mendelian disorder.

**Conclusions:**

We aim at providing a resource for the genetic disease community to automatically analyse whole exome-sequencing samples with a standard and uniform analysis pipeline, thus collecting variant allele frequencies by disorder. This resource may become a valuable tool to help dissecting the genotype underlying the disease phenotype through an improved selection of putative patient-specific causative or phenotype-associated variations.

## Background

Mendelian disorders are inherited diseases caused by inborn defects in the DNA sequence of one or few genes. Most inherited genetic disorders are rare, although if taken collectively, they are estimated to affect ~4% of newborns. There are ~7000 disease phenotypes described in the Online Mendelian Inheritance in Man (OMIM) Database [1] but the cause of about half of the described diseases is still unknown [2]. Whole Exome Sequencing (WES) of patients allows to find causative mutations of genetic diseases thanks to High-Throughput Sequencing (HTS) technologies [3]. WES is an effective alternative to standard genetic screenings to find causative mutations of genetic diseases when only few patients are available, as it is often the case for Mendelian disorders [4]. When compared to Whole Genome Sequencing (WGS), WES is still to be preferred because the targeted region comprises only 1-2% of the genome sequence and thus much less reads are required to get the sequencing depth necessary to reliably identify mutations. Furthermore, the potentially damaging effect of a coding-region mutation on the gene product activity can be predicted with good accuracy [5-10], but this is much more difficult in the case of a non-coding region mutation [11,12].

WES has been successfully used to find candidate causative mutations with as low as one affected individual [13-18]. One limitation of WES is that the percentage of samples where a candidate causative mutation is not found is still high [19]. This may happen when the causative mutation lies outside the targeted region or in a position difficult to sequence, or may be due to incomplete penetrance and the presence of modifier genes [20,21]. Another factor affecting the outcome of the analysis is the bioinformatic analysis pipeline [22] and its stringency level, since no standard operating procedure is currently available. This means that in order to compare results of different WES samples, it is important to use a uniform analysis pipeline and a common reference databases to prioritise the detected variants.

Indeed, despite the ever decreasing cost of sequencing experiments, the bioinformatic analysis of WES data requires high computational resources, trained experts and a reference variant database to select and prioritise the best candidate pathogenic variants.

Our aim was to build a community-based resource providing a disease-oriented allele variant frequency repository for Mendelian disorders populated by means of an automatic exome-sequencing analysis pipeline. The expansion and usefulness of this resource will be driven by user-submitted WES samples collected from Mendelian disorder patients.

## Implementation

### Website

**Figure 1:**
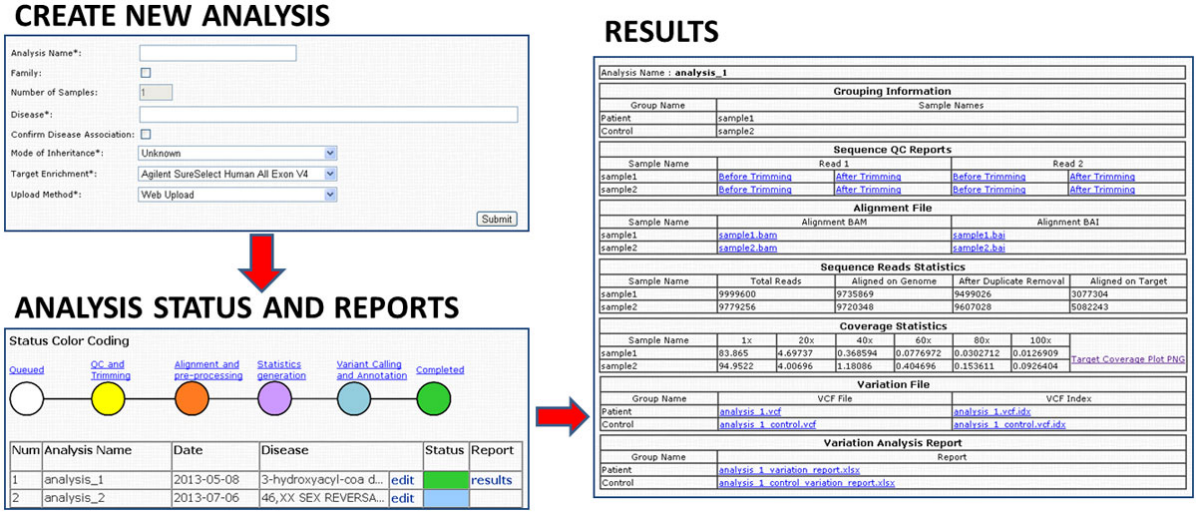
**Website interface.**Screenshots of the relevant sections of the web interface and outline of the user experience. The analysis is submitted through the *Create New *section, where the user submit the required analysis details. The user can choose to submit a single sample or multiple samples of related individuals and an auto-completion feature helps the user in assigning the correct disease id to the analysis. The user can follow the progress of the analysis in the *Analysis *section where he will also find a link to the Results page after the analysis is completed.

The website is implemented in *PHP*. After user registration, a new analysis can be started through the *Create New *submission page (Figure 1). The user has to provide the presumptive (or known) Mendelian disorder associated to the sample, the mode of inheritance and the platform used for exome target enrichment. The disease has to be chosen using a fixed vocabulary implementing the MEDIC hierarchical disease ontology [23] including all child terms to MeSH ID D009358: "Congenital, Hereditary, and Neonatal Diseases and Abnormalities". The disease list can be searched by directly typing the specific OMIM ID [1] or a keyword and the auto-completion function will automatically retreive all the available matching terms. The user should choose the definition that best describes the patient phenotype. The disease association can be later edited, for example when an initially presumptive diagnosis is then confirmed following the WES analysis. In such cases, the user will initially choose a less specific disease definition, using the controlled vocabulary, and can then change it to a more specific one after receiving the analysis results. Ideally, the user should confirm the diagnosis only after having validated the mutations found. The user can submit multiple samples at once, if the samples correspond to related individuals. Each sample has to be uploaded as a pair of sequence files in FastQ format [24]. The user can follow the analysis progression online and retrieve the results upon analysis completion (Figure 1).

### Pipeline Implementation

The analysis pipeline is fully automated and it has a modular structure, as detailed below and in Additional file 1. Each module performs its task using custom scripts and state-of-the-art tools (Additional file 2). The pipeline was designed to run on a high-performance computing cluster using the Torque resource manager, but can easily be ported to any other job manager. The exome.tigem.it website uses a cluster with 8 computing nodes equipped with dual Xeon E5-2670 for a total amount of 128 computing cores and 376GB of RAM.

#### Read quality assessment and trimming module

Read sequences are submitted by the user in FastQ format [24] and are initially assessed for the general quality using FastQC [25]. Reads are then trimmed to remove the Illumina adapter sequence and low quality ends (with quality score threshold of 20) using Trim Galore [26] and cutadapt [27]; a FastQC report is generated also on the trimmed sequences.

#### Alignment on reference, post-alignment processing and summary statistics Modules

Paired sequencing reads are aligned to the reference genome (UCSC, hg19 build) [28] using BWA [29]. Post-alignment process, including SAM conversion, sorting and duplicate removal are performed using Picard [30] and SAMtools [31]. The Genome Analysis Toolkit (GATK) [32] is then used to prepare the raw alignment for the variation calling with local realignment around small insertions-deletions (INDELs) and Base Quality Score Recalibration. This module is followed by a small module computing the read summary, target enrichment and target coverage statistics with SAMtools and BEDTools [33].

#### SNVs and INDELs calling and annotation Module

The identification of Single Nucleotide Variants (SNVs) and INDELs are separately performed using GATK UnifiedGenotyper, followed by Variant Quality Score Recalibration [34] when applicable. The SNV and INDEL calls are then merged and annotated using ANNOVAR [35] to add the following information: the position in genes and amino acid change relative to the RefSeq gene model [36], presence in dbSNP [37], OMIM [1], frequency in NHLBI Exome Variant Server [38] and 1000 Genomes Project stratified by population [39], prediction of the potential damaging effect on protein activity with different algorithms [5-10] and evolutionary conservation scores [40,41]. The annotated results are then imported into the variation database.

### Variation database and report generation module

The variation database is implemented in PostgreSQL and its structure with the main tables and relationships is shown in Additional file 3. A *variations *table contains an entry for each variation progressively collected in the database, each uniquely identified by genomic coordinates, reference and alternative alleles. Separate tables collect the statistics of the analysis calls, the annotation, the analysis and samples details. Finally, the *diseases *table contains the MEDIC hierarchical disease terms [23]. Once all the detected variants have been imported, the report generation module creates a report including all the variations found in the samples accompanied by the available annotations. Importantly, this module also dynamically computes allele frequencies stratified by disease groups, using the hierarchical disease ontology. In this way, even if no or few samples are available in the database for a specific Mendelian disorder, a sufficient number of samples can be reached by grouping samples at the higher levels of the disease ontology. The variation reports of all the archived analysis are periodically refreshed to update allele frequencies on the analyses gradually added to the database.

## Results and discussion

We developed a variation database for Mendelian disorders and associated WES analysis pipeline, in order annotate and store insertions, deletions and single nucleotide variants found in targeted resequencing projects, with a focus on patients affected by Mendelian disorders. The pipeline automates the analysis workflow using state-of-the-art tools, starting with raw sequences and providing the final list of annotated variants found in the sample. The pipeline allows for the simultaneous analysis of multiple samples of related individuals. This option is recommended when analysing members of the same family, who are expected to share the same causative mutation. In this case, the variant calling algorithm uses a multi-sample model that takes into account the global allele count in calling the individual genotypes, which can highly improve sensitivity [34]. It is also possible to analyse unaffected members of the family indicating them as controls. In this case the variants called in the unaffected members can be directly used to filter out all shared mutations that are not relevant in causing the proband phenotype.

This resource is complementary to free and commercial databases of known mutations associated to specific diseases or phenotype, such as the HGMD [42] or the ClinVar [43] databases or locus specific databases (LSDBs) [44], since it focuses on patients affected by Mendelian disorder. It is also different from the other large scale databases providing population frequencies because the collected samples are not phenotypically *normal*. Moreover, the associated WES analysis pipelines here presented has to be considered only as an accompanying tool to uniformly populate the database and cannot be considered a general purpose exome analysis pipeline, such as those recently presented in the literature [45-47].

The aim of this resource is to provide a standardised analysis of WES samples by providing state-ofthe-art pipeline and a standardised output of the variant calls and annotations, including the relative allele frequency in the *anonymised *samples already analysed in the database, stratified by disease.

Uniformity of the calling quality is ensured by analysing all samples with the same pipeline. The analysis was implemented to have a low stringency for the initial variant calling, in order to minimise the false negatives, but it relies heavily on intersection filters for controls and general population frequency to rule out non-causative mutations.

### Submission of whole exome sequencing samples

**Figure 2:**
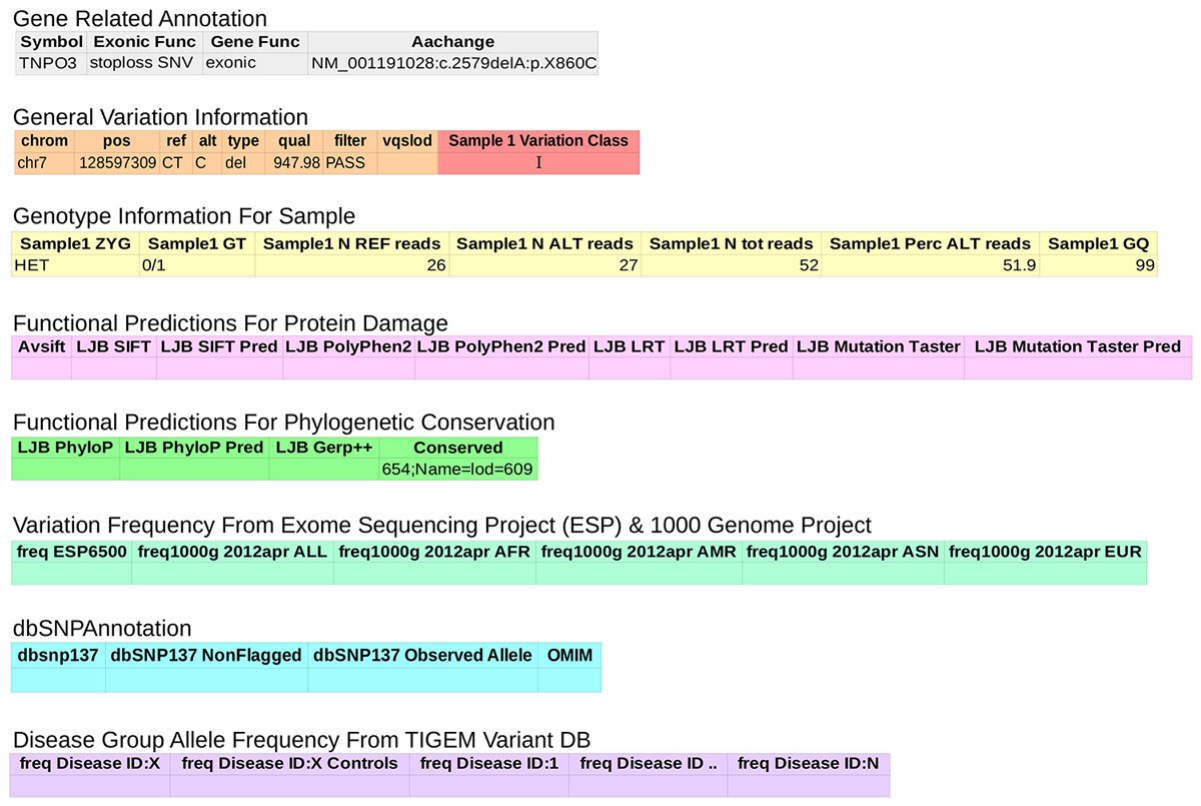
**Sample analysis report**. The analysis report produced by the analysis pipeline includes these fields for each variant called. In addition to the standard predictions of the variant on protein function, the report includes variant frequencies across patients grouped by disease according to the MEDIC hierarchical disease ontology. For a description of each field please refer to the Additional file 4.

Whole exome sequencing samples are submitted through a webpage http://exome.tigem.it shown in Figure 1. The user has to provide the required information about the analysis and the samples to be analysed and upload the sequences (in FastQ [24] format). Samples are required to be annotated with OMIM ID or, if a clear diagnosis is not available, with a MeSH term [48]. The analysis pipeline uses this annotation to group samples by disease and to calculate allele frequencies within disease groups (see Implementation). The analysis can be run on multiple samples provided they are from the same family and associated to the same disease (or associated controls, e.g. unaffected relatives). The user can check the analysis progress through the *Analysis *section where all the submitted analyses are archived. In the same section the Results page becomes available after the analysis was successfully completed. The Results page includes the files produces at several steps: the quality reports, the processed alignment in BAM format [31], reads and target coverage statics, the complete call results in vcf format [49] and the annotated table of variants (Figure 2). The user will find on the website notification of every annotation database updated or a major analysis pipeline improvement and can choose to download updated results. Importantly, the sequence data (i.e. FastQ and BAM files) will never be made public, and on request these files will be deleted from the servers (as specified in the online User agreement). In this case, however, the user will not be able to get updated results.

### Automated analysis workflow

As detailed in the Implementation section, the pipeline workflow follows a state-of-the-art implementation of the exome sequencing analysis [50] (Additional file 1). The analysis is initialized by a master script that configures and submits the modules performing the actual analysis steps on the computing cluster. The modules are configured with pre-defined sets of parameters to ensure uniformity of sensitivity across analyses. The user can only choose the number of samples to analyze, either as a single case or as a group analysis by selecting the Family option. In this latter case, also control samples are allowed, but these are analyzed separately.

The first module in the pipeline performs a quality assessment of reads and trimming of read ends to remove the adapter sequence or trailing low quality bases. Then reads are aligned to the reference genome (UCSC hg19 [28]) and the alignment is prepared for variation calling trough a series of steps: format conversion, sorting, local realignment around INDELs and Base Quality Score Recalibration. The local realignment around INDELs is an important step. It finds a consensus alignment among all the reads spanning a deletion or an insertion to both improve INDEL detection sensitivity and accuracy and to reduce SNV false calls due to misalignment of the flanking bases. The Base Quality Score Recalibration is a procedure through which the raw quality scores provided by the instrument are recalibrated according an empirical error model derived by the sequences [34]. The SNV and INDEL variant calling are then performed and the calls are merged and annotated with information collected from several sources (Figure 2). The pipeline is designed to run on a cluster and can submit jobs in parallel to analyse several samples simultaneously. The annotated variant calls are then imported into the variant database.

### Variant annotation and reporting

The variation database is used to store the annotated exonic/splicing variants and to calculate allele frequencies stratified by groups of patients presenting the same, or similar, disease or phenotype according to the OMIM identifiers and MeSH terms, implementing the MEDIC hierarchical disease ontology [23]. Importantly, the internal allele frequency among samples progressively collected in the database itself, stratified by Mendelian disorder, is estimated, thus leading to a better selection of putative disease-specific causative variations.

The database includes also annotations of variants from external sources (e.g. dbSNP, 1000genomes, Exome Variant Server and prediction algorithms), which are stored in a separate table and are periodically updated upon release of a new version of one or more external source database.

The final report of the analysis, which will be available to the user, is a Microsoft Excel file including a table with all the relevant information useful to filter the selected variants and to prioritise them in order to choose the best possible candidates for subsequent validation (Figure 2). Specifically, in order to help the user in the filtering process, the table classifies variants in five classes, as shown in Table 1, on the basis of three factors: frequency in the general population, in unrelated diseases, and in the same or related disease(s), quality of the call and predicted impact on the gene product activity.

**Table 1:** Variation Classification

Variation Class			
**Class **	**Frequency **	**Quality **	**Impact **

I	+	+	+

II	+	+	-

III	+	-	+

IV	+	-	-

V	-	+/-	+/-

Variants are automatically classified by the pipeline to help the user in detecting causative mutations (also refer to Additional file 4). A "+" sign means that the criterion indicated in the column is satisfied. Frequency criterion: the frequency in 1000 Genomes Project, Exome Variant Server and the TIGEM Variant database (in unrelated disease groups) must be < 1%; Quality criterion: the GATK variant calling tool must assign a "PASS" value to the "filter" field and score > 50 to the Genotype Quality field. Moreover, if the variant is homozygous, the percentage of reads supporting the call must be > 80%. If the variant is heterozygous, the percentage of reads supporting the call must be > 30% and < 80%); Impact criterion: the mutation causes a gain or loss of a stop codon, a gain or loss of a splicing signal, or a frame-shift in the Open Reading Frame. Alternatively, the phylogenetic conservation must be significant (i.e. the fields LJB PhyloP Pred="C" AND LJB Gerp++>2 AND Conserved>170) and 4 out of 5 prediction algorithms indicate a damaging effect (i.e. Avsift, LJB SIFT, LJB PolyPhen2, LJB LRT, LJB Mutation Taster).

We give priority to the frequency criterion since when dealing with rare Mendelian disorders it is unlikely that the causative mutation may be common in the general population. These categories should be regarded as guides in prioritising the variant called in the analysis and can help in quickly highlighting the best candidate(s).

## Conclusion

We developed a resource for the analysis of WES samples for researchers studying Mendelian disorders. We believe this resource will be useful not only for those who do not have the hardware resources or the necessary expertise to run the analysis, but, more importantly, as a common reference for the community to collect and compare variants across patients with the same, or similar, disease.

Each researcher by submitting data to the resource will enrich the database and thus leverage the frequency of the variations potentially associated to the Mendelian diseases. For this reason, we require all samples to be annotated with the OMIM/MeSH corresponding to the patient phenotype in order to update the corresponding group allele frequencies with the new samples variant calls.

The analysis report classifies variation by classes to help the user in prioritising candidate mutants. These classes should be regarded as prioritising guides and not as hard filters because it is possible that low-quality calls (e.g. due to low coverage or other technical problems in the regions) are true mutations that can be validated and could be lost in a highly stringent analysis.

The resource provides variant frequencies according to disease groups, thus helping in detecting modifier or secondary mutations which tend to be more represented in the patients affected by the same phenotype. The estimation of statistically significant associations will improve with the number of patients with homogeneous phenotype collected in the resource.

The TIGEM Exome Mendelian Disorder Pipeline is a new community-based resource available to the Mendelian diseases research community, built with the aim of help in dissecting the genotype underlying the disease phenotype in patients affected by rare diseases.

## Availability and requirements

• *Project name: TIGEM Exome Mendelian Disorder Pipeline*

• *Project home page: http://exome.tigem.it*

• *Operating system(s): Platform independent*

• *Programming language: bash, perl, R, SQL, PHP*

• *License: Terms of use are on the website*

## List of abbreviations

BAM: Binary Alignment Map; GATK: Genome Analysis Toolkit; HTS: High-Throughput Sequencing; INDEL: small insertion or deletion; NGS: Next Generation Sequencing; SNP: Single Nucleotide Polymorphism; SNV: Single Nucleotide Variation; WES: Whole Exome Sequencing; WGS: Whole Genome Sequencing; VCF: Variant Call Format.

## Competing interests

The authors declare that they have no competing interests.

## Authors' contributions

MM and VSM designed and developed the analysis pipeline. DC developed the web interface and built the variation database. RR contributed to the pipeline and building of the database. GD participated in the initial design of the analysis workflow. GO helped in developing the pipeline and the web interface. MM and DdB supervised the project development. DdB conceived the idea. MM, VSM, DC and DdB drafted the manuscript. All authors read and approved the final manuscript.

## Supplementary Material

Additional file 1 Additional Figure 1. Pipeline workflow scheme. The Analysis Master represents the main wrapper script that reads input parameter and creates a new sample analysis in the Configuration DB. The parameters stored in the Configuration DB are then passed to the individual modules, represented in blue, here grouped according to different phases of analysis representing the main steps. The results are imported into the TIGEM Variant DB, which stores all variant and annotation information. The TIGEM Variant DB is then queried to generate the final report. The files delivered to the end user are marked with a red colored asterisk. Click here for file

Additional file 2 Additional Table 1. Analysis tools implemented in the pipeline. List and current version of the analysis tools used in the pipeline. Click here for file

Additional file 3 Additional Figure 2. Variation Database structure. Scheme of the main tables and relationships in the Variation Database. Click here for file

Additional file 4 Additional Table 2. Analysis report column legend. Legend of the representative fields in the analysis report. Click here for file

## Declarations

The publication costs for this article were funded by the Italian National Research Center (CNR -Flagship Project EPIGEN).

This article has been published as part of *BMC Genomics *Volume 15 Supplement 5, 2014: Italian Society of Bioinformatics (BITS): Annual Meeting 2013: Genomics. The full contents of the supplement are available online at http://www.biomedcentral.com/bmcgenomics/supplements/15/S3
