# Supplementary figures and images for: A community-based resource for automatic exome variant-calling and annotation in Mendelian disorders
Source: BMC Genomics. 2014 May 6;15(Suppl 3):S5. doi: 10.1186/1471-2164-15-S3-S5 (PMC4083405; doi:10.1186/1471-2164-15-S3-S5)

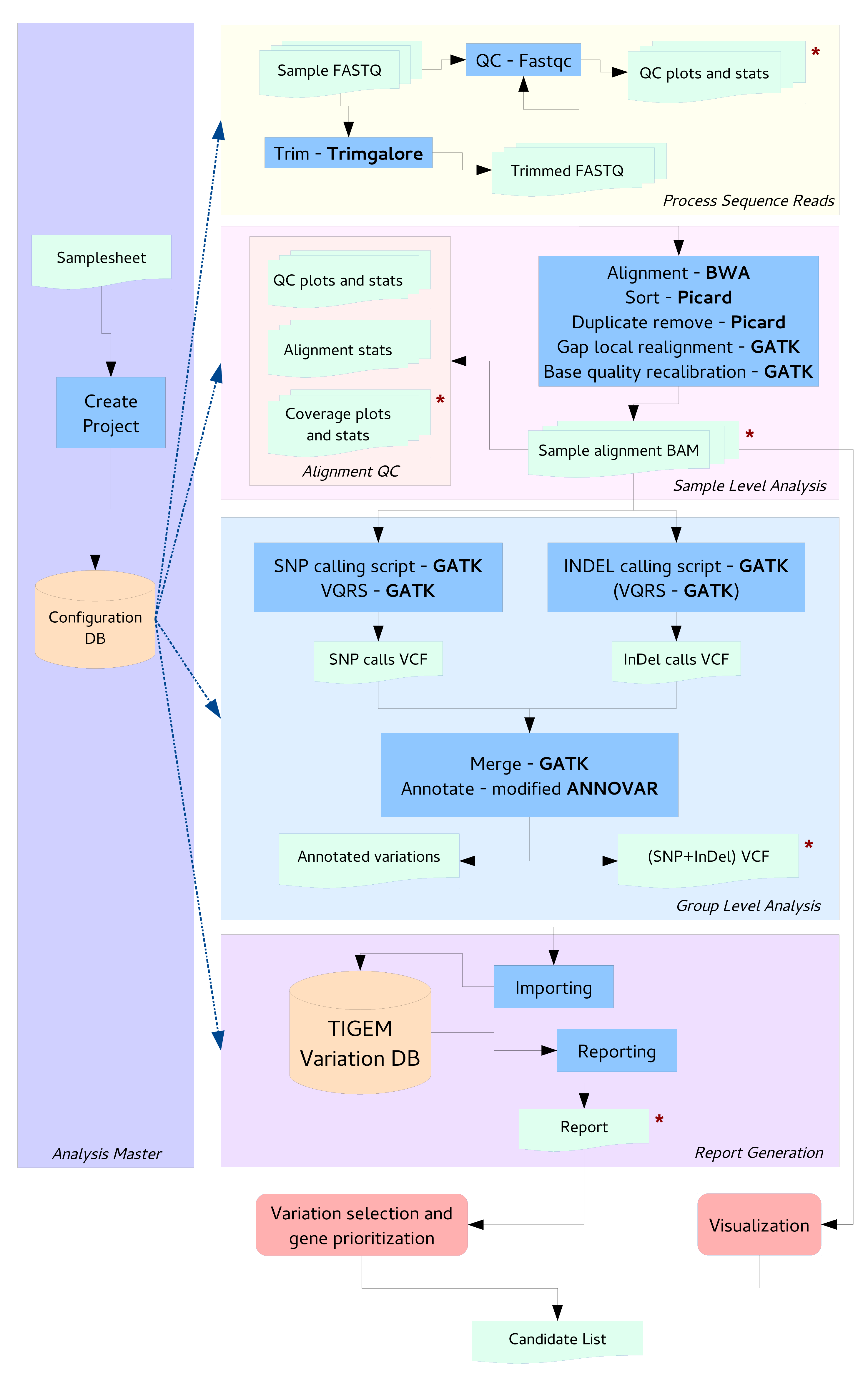

Supplement: Additional file 1 — Additional Figure 1. Pipeline workflow scheme. The Analysis Master represents the main wrapper script that reads input parameter and creates a new sample analysis in the Configuration DB. The parameters stored in the Configuration DB are then passed to the individual modules, represented in blue, here grouped according to different phases of analysis representing the main steps. The results are imported into the TIGEM Variant DB, which stores all variant and annotation information. The TIGEM Variant DB is then queried to generate the final report. The files delivered to the end user are marked with a red colored asterisk. [file 1471-2164-15-S3-S5-S1.png]

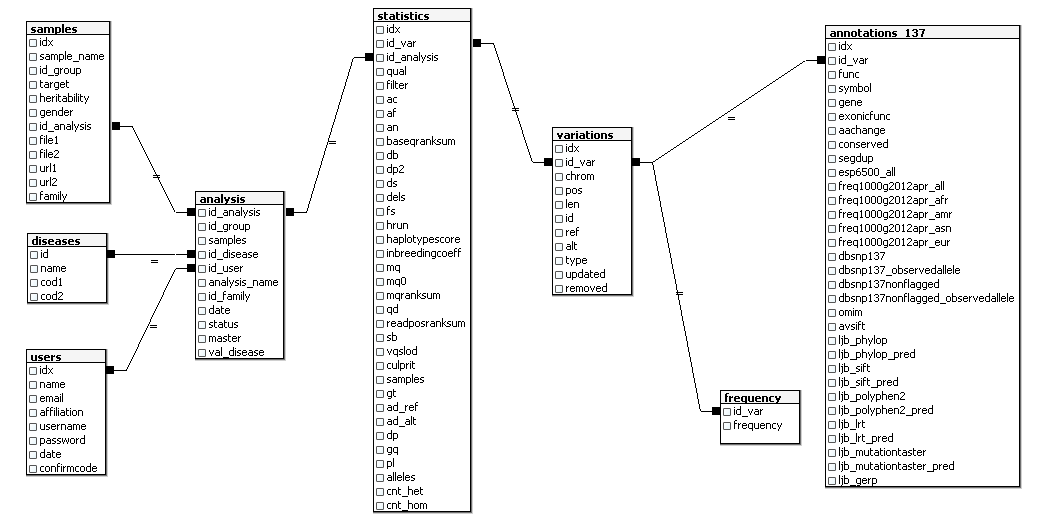

Supplement: Additional file 3 — Additional Figure 2. Variation Database structure. Scheme of the main tables and relationships in the Variation Database. [file 1471-2164-15-S3-S5-S3.png]
