# Supplementary material for: A community-based resource for automatic exome variant-calling and annotation in Mendelian disorders
Source: BMC Genomics. 2014 May 6;15(Suppl 3):S5. doi: 10.1186/1471-2164-15-S3-S5 (PMC4083405; doi:10.1186/1471-2164-15-S3-S5)
Supplement: Additional file 2 — Additional Table 1. Analysis tools implemented in the pipeline. List and current version of the analysis tools used in the pipeline. [file 1471-2164-15-S3-S5-S2.pdf]

Analysis Tools		
**Name**	**Version**	**Processes**
FASTQC	0.10.1	Sequence QC
Trim Galore	0.3.1	Sequence Trimming
BEDtools	v2.16.2	Statistics
BWA	0.6.2	Sequence Alignment
SAMtools	0.1.19	Alignment Statistics
Picard	1.81	Alignment Processing and Statistics
GATK	2.7-4	Variation Calling, Alignment Processing and Statistics
Annovar	2013May20	Annotate Variations
