# Supplementary material for: A community-based resource for automatic exome variant-calling and annotation in Mendelian disorders
Source: BMC Genomics. 2014 May 6;15(Suppl 3):S5. doi: 10.1186/1471-2164-15-S3-S5 (PMC4083405; doi:10.1186/1471-2164-15-S3-S5)
Supplement: Additional file 4 — Additional Table 2. Analysis report column legend. Legend of the representative fields in the analysis report. [file 1471-2164-15-S3-S5-S4.pdf]

Name	Description
**Symbol**	Gene Symbol
**Exonic Func**	Feature location of the variant (exonic, splicing)
**Gene Func**	Functional consequences of the variant (nonsynonymous SNV, synonymous SNV, frameshift insertion, frameshift deletion, nonframeshift insertion, nonframeshift deletion, frameshift block substitution, nonframshift block substitution)
**Aachange**	Amino acid change
**chrom**	Chromosome name
**pos**	Chromosomal position
**ref**	Reference allele
**alt**	Alternative observed alleles
**type**	SNV or INDEL
**qual**	Global score of the calling
**filter**	Result of the global quality filter after the gaussian mixture model: 'PASS'=passed
**vqslod**	Log odds ratio of being a true variant versus being false under the trained gaussian mixture model
**Variation Class**	Classification of the variation (I,II,IIIIV,V)
**<sample name> ZYG**	Decoded genotype
**<sample name> GT**	Called genotype
**<sample name> N REF reads**	N reads supporting the reference allele
**<sample name> N ALT reads**	N reads supporting the alternative allele
**<sample name> N tot reads**	Total number of reads at position
**<sample name> Perc ALT reads**	Percentage of reads supporting the alternative allele
**<sample name> GQ**	Genotype Quality
**Avsift**	SIFT pre-calculated P-value: if < 0.05 the mutation is considered deleterious
**LJB SIFT**	SIFT from dbNSF (between 0 and 1, the higher the more deleterious)
**LJB PolyPhen2**	PolyPhen2 from dbNSF (between 0 and 1, the higher the more deleterious)
**LJB LRT**	LRT from dbNSF (between 0 and 1, the higher the more deleterious)
**LJB Mutation Taster**	MutationTaster from dbNSF (between 0 and 1, the higher the more deleterious)
**LJB PhyloP**	PhyloP conservation score
**LJB PhyloP Pred**	PlyloP prediction: C (conserved) if score > 0.95, otherwise N (non conserved)
**LJB Gerp++**	Gerp++ conservation score (the higher the more conserved)
**Conserved**	Conservation score from UCSC 46 species alignment: range 0-1000, the higher the more conserved
**freq ESP6500**	Frequency in NCBI 6500 exomes project
**freq1000g 2012apr ALL**	Frequency in 1000 genomes data (release apr 2012), all populations together
**freq1000g 2012apr AFR**	Frequency in 1000 genomes data (release apr 2012), subpop
**freq1000g 2012apr AMR**	Frequency in 1000 genomes data (release apr 2012), subpop
**freq1000g 2012apr ASN**	Frequency in 1000 genomes data (release apr 2012), subpop
**freq1000g 2012apr EUR**	Frequency in 1000 genomes data (release apr 2012), subpop
**dbsnp137**	dbSNP 137 ID
**dbSNP137 NonFlagged**	dbSNP 137 after removing those flagged SNPs (SNPs < 1% minor allele frequency (MAF) (or unknown), mapping only once to reference assembly, flagged in dbSNP as "clinically associated")
**dbSNP137 Observed Allele**	dbSNP 137 Observed Allele
**OMIM**	OMIM ID
**freq OMIM:X**	Allele frequency in patients affected by disease OMIM:X
**freq OMIM:X Controls**	Allele frequency in unaffected controls of disease OMIM:X
**freq OMIM 1**	Allele frequency in patients affected by disease OMIM ID1
**freq OMIM ..**	Allele frequency in patients affected by disease OMIM ID…
**freq OMIM:N**	Allele frequency in patients affected by disease OMIM:N
